# Anatomical landmarks of the intra-pelvic side-wall as sources of pain in women with and without pregnancy-related chronic pelvic pain after childbirth: a descriptive study

**DOI:** 10.1186/s12905-018-0542-z

**Published:** 2018-03-27

**Authors:** Thomas Torstensson, Stephen Butler, Anne Lindgren, Magnus Peterson, Lena Nilsson-Wikmar, Margaretha Eriksson, Per Kristiansson

**Affiliations:** 1 Department of Public Health and Caring Sciences, Uppsala University, Box 564, 751 22 Uppsala, SE Sweden; 2 Departmemt of Physiotherapy, Sundsvall Hospital, 851 86 Sundsvall, SE Sweden; 3 Pain Center, Uppsala University Hospital, 751 85 Uppsala, SE Sweden; 4 Departmemt of Neurobiology, Care Sciences and Society, Division of Physiotherapy, Karolinska Institutet, 141 83 Huddinge, SE Sweden; 5 Academic Primary Healthcare Centre, Stockholm County Council, 104 31 Stockholm, SE Sweden

**Keywords:** Anatomical landmarks, Intra-pelvic side-wall, Chronic pelvic pain, Pregnancy-related

## Abstract

**Background:**

Chronic pelvic pain (CPP) affects 15–24% of women and can have a devastating impact on quality of life. Laparoscopy is often used in the investigation, although in one third of the examinations there is no visible pathology and the women may be dismissed without further investigation. Also, the contribution of skeletal, muscular, periosteal and ligamentous tissues to CPP remains to be further elucidated. The objective of the present study was to compare pain intensity provoked from anatomical landmarks of the intra-pelvic side-wall in women with pregnancy-related CPP after childbirth and women without such pain.

**Methods:**

This is a descriptive study of 36 non-randomly selected parous women with CPP after childbirth and 29 likewise selected parous women after childbirth without CPP. Pain was determined by questionnaire and clinical examination. The primary outcome measure was reported pain intensity provoked on 13 anatomical landmarks of the intra-pelvic side-wall. All women reported their perceived pain intensity for each anatomical landmark on Likert scales and an individual sum score was calculated.

**Results:**

Women with chronic pelvic pain were older than women without CPP. At several intra-pelvic landmarks high intensity pain was provoked in women with CPP compared with less intense pain provoked at fewer landmarks in women without low back or pelvic pain (*p* < 0.0001). The average sum of pain intensity scores was about 4 times higher in women with CPP (1.3) as compared with those without low back or pelvic pain (0.3), *p* < 0.0001. This association remained when adjusting for the age difference between the pain groups in linear regression analysis. In addition, reported pain intensity at worst past week was independently associated with sum of pain intensity scores. The maximum individual sum of pain intensity scores among women without CPP was exceeded by that of 85% of the women with CPP.

**Conclusions:**

Parous women with CPP after childbirth had a heightened pain intensity over 13 anatomical landmarks during pelvic examination compared with parous women without CPP. These results need to be confirmed in a larger cohort with different types of CPP.

## Background

Chronic pelvic pain (CPP) is a significant problem for both General Practitioners and Gynecologists. Chronic pelvic pain affects 15–24% of women and can have a devastating impact on quality of life. The pain syndrome is costly for both the individual and health service providers [1, 2]. Diagnosis and localization of pelvic pain symptoms to specific pain generators are a challenge made difficult possibly due to the convergence of afferent input from both somatic (including scleral) and visceral tissues in central representations of the peripheral nervous system [3].

Given the large burden for the individual and society, mechanism-based treatment is desirable. With this perspective, laparoscopy is often used in the investigation of women with chronic pelvic pain with the focus on finding visible gynecologic pathology suitable for medical or surgical intervention, such as endometriosis or other visceral disease [4]. However, if there is no visible pathology, as is the case in one third of laparoscopies due to chronic pelvic pain, or if pathology is present and treatment only partially effective or ineffective the women may be dismissed without consideration of further investigation or alternative treatment [5–7].

Also, improvement in diagnosis is an important objective because treatment strategies for pain arising from ligaments, joints, periosteum or myofascial structures differ from treatment strategies for, for example, endometriosis. Provocation tests can be used to diagnose chronic pelvic pain due to sacroiliac joint dysfunction but specificity has been questioned and extra-articular sources of pain have been suggested [8–15]. Pelvic floor muscles have been examined by vaginal palpation and found to be more sensitive to stretch in women with chronic pelvic pain than in healthy women [16–18]. Also, there is a correlation between provocation tests and myofascial structures as pain generators [19, 20]. However, the contribution of skeletal and ligamentous tissues to chronic pelvic pain remains to be further elucidated.

The aim of this study was to compare the intensity of pain provoked from anatomical landmarks of the intra-pelvic side-wall and to test its discrimination property between women with chronic pelvic pain after childbirth and women without low back and pelvic pain. The hypothesis was that the pain intensity provoked on intra-pelvic side-wall sites differed between the groups of women with and without chronic pelvic pain.

## Methods

This was a descriptive study with 36 non-randomly selected parous women with chronic pelvic pain after childbirth and 29 non-randomly selected parous women without low back or pelvic pain. The primary outcome measure was reported pain intensity provoked on anatomical landmarks of the intra-pelvic side-wall.

Thirty-six women, out of 36 requested, with chronic pelvic pain after childbirth were selected from a physiotherapy department waiting list and by advertisements in newspapers. This group has been described in detail elsewhere [21]. The inclusion criteria were: 1) reporting persistent pregnancy-related sacral pain for at least six months after childbirth, 2) pain intensity of at least 30 mm on a visual analogue scale where 0 mm is no pain and 100 mm is worst possible pain, 3) having one of Menell’s, Patrick’s or Posterior Pelvic Pain Provocation (P4) test positive, 4) having provoked ipsilateral pain at the ischial spine on vaginal palpation and 5) ability to understand Swedish. Exclusion criteria were: 1) reporting persistent low back or pelvic pain beginning before pregnancy, 2) previous back or pelvic surgery and 3) signs of neurological deficits (positive straight leg raising test or loss of a tendon reflex in a lower extremity).

In order to select a group of parous women without reported pain and no pain provoked by examination of the low back or pelvis externally, 44 women were consecutively selected after written informed consent. The source was women from the routine gynecologic control to which all women between 24 and 60 years of age are regularly invited for pap-smear tests. The site was a midwifery surgery in the Primary Health Care Centre in Sundsvall, Sweden. The women selected indicated any ongoing pain on a pain drawing and a physiotherapist (A.L.) performed an external physical examination of the low back and pelvis on all the 44 women. With this information the physiotherapist allocated 15 women to a group with ongoing pain in the low back or pelvis and 29 women to a group without such pain. All 44 women subsequently underwent a pain intensity provocation of anatomical landmarks of the intra-pelvic side-wall, as described later, by a physician (P.K.) blinded as to the allocation group. Finally, the 29 women in the no pain group were included in the study.

**Fig. 1:**
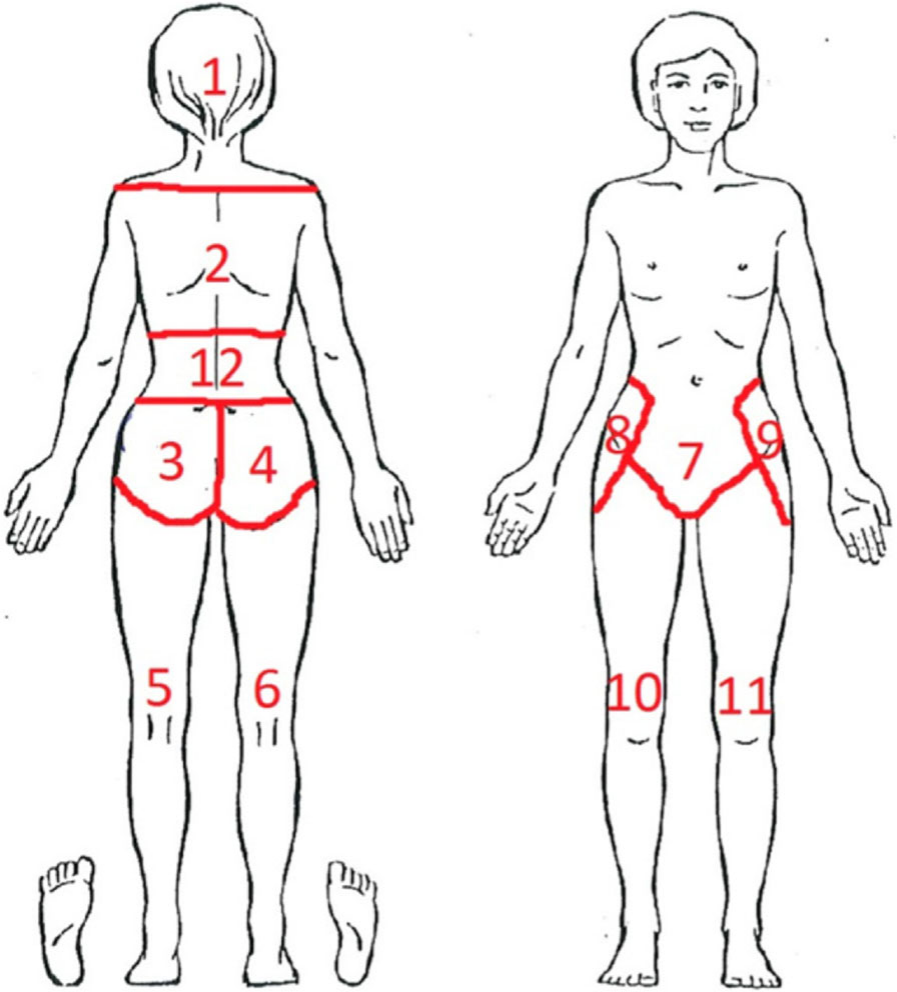
Pain drawing to report location of any on-going pain. The pain locations were coded as: 1) head, 2) thoracic spine, 3) sacral area left, 4) sacral area right, 5) leg back left 6) leg back right, 7) symphysis and low abdomen 8) hip right, 9) hip left, 10) leg front right, 11) leg front left and 12) lumbar spine

The physiotherapist’s evaluation before allocation included information collected from a questionnaire and from the external physical examination of the low back and pelvis. The questionnaire included a pain drawing of the body where the women indicated any pain location and these were subsequently coded according to Fig. 1. The number of pain locations was then summed. In addition, the questionnaire included information about the time of onset of ongoing low back or pelvic pain, number of previous pregnancies and deliveries, date of latest delivery, smoking habits at present (no/yes), level of education (≤12 years/> 12 years) and previous back or pelvic surgery. The women were also requested to report pain intensity at present and worst pain during the past week using a visual analogue scale, which ranged from 0 (no pain) to 100 mm (worst possible pain) [22]. They also completed the Disability Rating Index questionnaire, an instrument for self-reported physical function (0–100 mm) where lower values represent higher function [23]. The physical examination of the low back included pain provocation tests of maximum flexion/extension while standing, which were considered positive if pain was elicited anywhere throughout the range of movement. Also included was palpation of the paravertebral region of the low back and iliolumbar ligament, bilaterally, which were considered positive if pain was elicited with moderate pressure. The pain provocation tests on the pelvis were Menell’s, Patrick’s and Posterior Pelvic Pain Provocation, which were performed in the supine position and considered positive if an aggravated ipsilateral sacral (buttocks included) pain was elicited; otherwise negative [24, 25]. In addition, Achilles and patellar tendon reflexes were assessed and passive straight leg raising was tested in each leg with the women in the supine position and was considered positive if neurological symptoms occurred or radiating pain was provoked [26]. To be allocated to the low back or pelvic pain group the inclusion criteria were: reported pain in the areas 3, 4, 7, 8 or 9 (according to Fig. 1), any low back or pelvic pain provocation test positive or positive neurologic tests. Otherwise the women were allocated to the no pain group.

### Pain intensity provocation of anatomical landmarks of the intra-pelvic side-wall

**Fig. 2:**
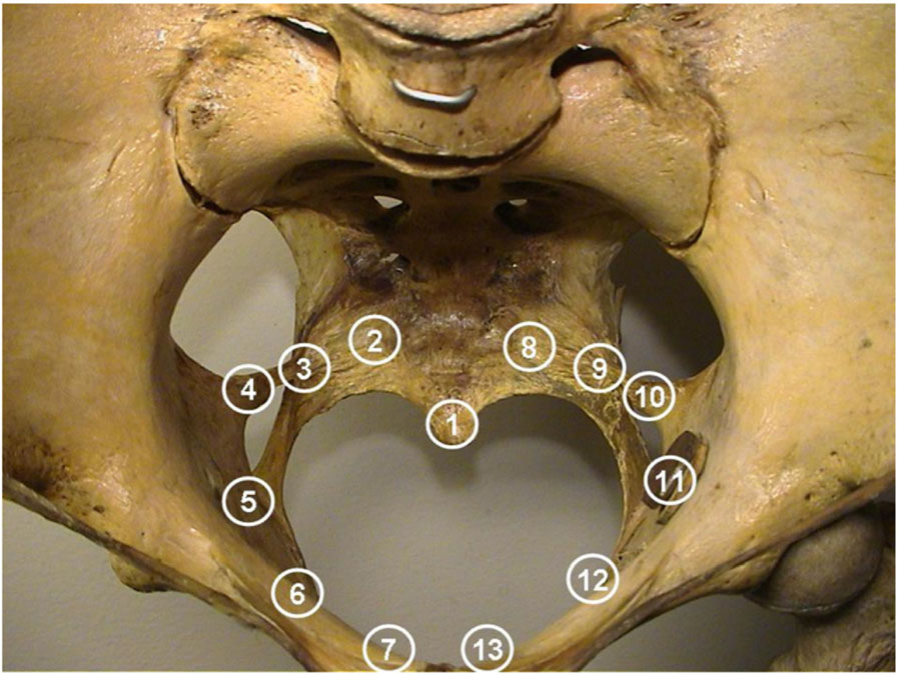
Anatomical landmarks of the intra-pelvic side-wall: 1) os coccyx, 2 and 8) os sacrum laterally, 3 and 9) sacrospinous ligament, 4 and 10) ischial spine, 5 and 11) os ischium inferior to the ilio-ischial fusion, 6 and 12) os pubis laterally and 7 and 13) os pubis medially

All women enrolled in the study were examined by pain intensity provocation of the intra-pelvic side-wall. The pain intensity provocation was performed by light manual pressure on each of 13 predetermined anatomical landmarks of the intra-pelvic side-wall by vaginal palpation with the women in the supine lithotomy position without stirrups. The light manual pressure corresponded to the pressure that produced blanching of the nail bed. The women were asked to report the perceived pain intensity on each of the landmarks on a Likert scale: 0) no pain, 1) moderate pain and 2) intensive pain. An individual sum score of pain intensity elicited on all 13 anatomical landmarks was calculated (range 0 to 26). The landmarks represented possible pain sources of anatomically distinct ligamentous or skeletal structures of the pelvic side-wall. The chosen anatomical landmarks were: the coccyx, the lateral part of sacrum at the insertion of the sacrospinous ligament, the middle part of the sacrospinous ligament, the insertion of the sacrospinous ligament at the ischial spine, the ischium inferior to the ilio-ischial fusion and the lateral and medial part of the pubic bone, Fig. 2. Except the coccyx, the landmarks were examined bilaterally and in the same order. A physiotherapist (T.T.) aided in the recording of the provoked pain intensity.

### Statistical analysis

Summary statistics were computed using standard methods and presented as means and standard deviations. For the simple and multiple linear regression analyses the scale assigned for categorization of ordinal factors used in the model was no chronic pelvic pain/chronic pelvic pain. No missing value imputation was performed. Only two-tailed tests were used. A value of *p* < 0.05 was regarded as significant. Very low *p*-values were indicated as < 0.0001. Statistical analysis was performed using the SAS program package version 9.3 (SAS Institute, Cary, NC).

## Results

Women with chronic pelvic pain were older than women with no low back or pelvic pain, but no differences regarding number of previous pregnancies and deliveries, proportion of smokers and education > 12 years, Table 1. All pain and disability reports were higher in those with chronic pelvic pain as compared with those without.

**Table 1:** Characteristics of the included women grouped into those with pregnancy related chronic pelvic pain (CPP) after childbirth and those without low back or pelvic pain (No CPP). Means (s.d.) or numbers (%) are presented

Characteristic	CPP (*n* = 36)	No CPP (*n* = 29)	*p* ^*)^
Age (years)	32.8 (5.0)	42.1 (6.8)	< 0.0001
No. of previous pregnancies	2.6 (1.0)	2.6 (1.1)	0.83
No. of previous deliveries	2.2 (0.8)	2.1 (0.7)	0.88
No cigarette smoking (%)	32 (89)	24 (83)	1.00
Education > 12 years (%)	16 (44)	16 (55)	0.21
Pain intensity at present (mm)	38.3 (16.1)	2.7 (5.9)	< 0.0001
Pain intensity at worst past week (mm)	58.6 (22.9)	6.1 (12.3)	< 0.0001
No. of pain locations	6.4 (2.4)	0.7 (1.0)	< 0.0001
Disability rating index (mm)	50.7 (19.5)	4.8 (8.5)	< 0.0001

^*)^The p-value refers to the difference between the groups

Provoked pain intensity scores on the 13 anatomical landmarks of the intra-pelvic side-wall are shown in Table 2. All scores of pain intensity were higher among women with chronic pelvic pain as compared with women without low back and pelvic pain (*p* < 0.0001). In women with chronic pelvic pain, the anatomical landmark with the highest provoked pain intensity was the ischial spine, with a mean score of 1.7 (s.d. 0.4), from which the pain intensity successively decreased in both directions further away from the ischial spine (*p* < 0.0001). A similar trend of the provoked pain intensity between the anatomical landmarks was shown among women without low back or pelvic pain (0.002 < *p* < 0.17).

**Table 2:** Score of pain intensity (graded 0-1-2) provoked on 13 anatomical landmarks of the intra-pelvic side-wall, among women with pregnancy related chronic pelvic pain (CPP) after childbirth and those without low back or pelvic pain (No CPP). The average score of the symmetric landmarks and os coccyx and the average and sum scores of all 13 landmarks are presented as means (s.d.)

Anatomical landmark	CPP (*n* = 36)	No CPP (*n* = 29)	*p* ^*)^
Os sacrum laterally	1.1 (0.5)	0.2 (0.5)	< 0.0001
Sacrospinous ligament	1.3 (0.5)	0.3 (0.4)	< 0.0001
Ischial spine	1.7 (0.4)	0.4 (0.5)	< 0.0001
Os ischium	1.6 (0.5)	0.5 (0.4)	< 0.0001
Os pubis laterally	1.3 (0.5)	0.3 (0.3)	< 0.0001
Os pubis medially	1.2 (0.7)	0.2 (0.4)	< 0.0001
Os coccyx	1.2 (0.7)	0.2 (0.4)	< 0.0001
Average of all thirteen	1.3 (0.4)	0.3 (0.3)	< 0.0001
Sum of all thirteen	17.4 (5.0)	4.1 (3.4)	< 0.0001

^*)^The p-value refers to the difference between the women with and without CPP

The mean sum of pain intensity scores provoked on all 13 intra-pelvic landmarks was 17.4 (s.d. 5.0) in women with chronic pelvic pain and 4.1 (s.d. 3.4) in women without low back or pelvic pain (*p* < 0.0001). The average score of all thirteen landmarks was 4.3 times higher in women with chronic pelvic pain as compared with those without low back or pelvic pain (p < 0.0001). The distribution of the sum of pain intensity scores among women with chronic pelvic pain and women without low back and pelvic pain are shown in Fig. 2b. Provoked pain intensity scores on the 13 anatomical landmarks except the ischial spines displayed similar differences between the groups: the mean sum was 14.7 (s.d. 4.7) in women with chronic pelvic pain and 3.6 (s.d. 2.7) in women without low back or pelvic pain (p < 0.0001).

The sum of pain intensity scores among women with chronic pelvic pain was correlated with the reported pain during the past week (*r* = 0.39, *p* = 0.02) but not with age, number of previous pregnancies, number of previous deliveries, cigarette smoking, education level, pain intensity at present, number of pain locations or disability rating index. Among women without chronic pelvic pain no associations were displayed.

**Fig. 3:**
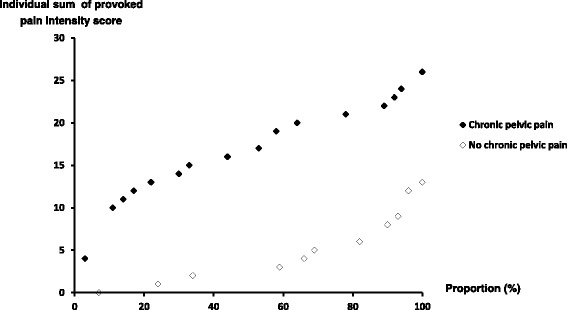
Cumulative proportion of the individual sum of provoked pain intensity scores of 13 anatomical landmarks of the intra-pelvic side-wall among women with pregnancy related chronic pelvic pain (*n* = 36) and women without low back or pelvic pain (*n* = 29)

The cumulative proportion of the sums of pain intensity scores provoked on all the 13 anatomical landmarks of the intra-pelvic side-wall across women with chronic pelvic pain and women without low back or pelvic pain is displayed in Fig. 3. The minimum and maximum of the sum score among women with chronic pelvic pain were 4 and 26 respectively and in women without low back or pelvic pain, 0 and 13. The highest score of 13 for women without low back or pelvic pain was exceeded by 85% of the women with chronic pelvic pain.

To investigate the impact of age for the sum of pain intensity scores among women with or without pelvic pain linear regression analysis was used, Table 3. Age was inversely associated with the sum score in the crude analysis, but did not remain significant when age or pain intensity at worst past week were introduced in the analyses. In the full model both pain group and pain intensity at worst past week were significantly associated with the sum score, with an R^2^ of 0.73.

**Table 3:** Score of pain intensity (graded 0-1-2) provoked on 13 anatomical landmarks of the intra-pelvic side-wall, among women with pregnancy related chronic pelvic pain (CPP) after childbirth and those without low back or pelvic pain (No CPP). The average score of the symmetric landmarks and os coccyx and the average and sum scores of all 13 landmarks are presented as means (s.d.)

	Crude			Model 1			Model 2		
Characteristic	Β-coeff	SE	*p*	β-coeff	SE	*p*	β-coeff	SE	*p*
Pain group (No CPP/CPP)	13.3	1.1	< 0.0001	13.66	1.40	< 0.0001	9.57	1.83	< 0.0001
Age (yr)	−0.52	0.12	< 0.0001	0.04	0.09	0.63	0.11	0.09	0.23
Pain intensity at worst past week (mm)	0.19	0.02	< 0.0001				0.09	0.03	0.002
Adjusted R^2^				0.69			0.73		

β = β-coefficient, SE = standard error. CPP = chronic pelvic pain

## Discussion

Parous women with CPP after childbirth have a heightened pain intensity over 13 anatomical landmarks during pelvic examination compared with parous women without CPP. This suggests that pain provocation by stimulation of intra-pelvic landmarks can be valuable in the clinical setting to discriminate women with chronic pelvic pain after childbirth from those without and to identify a subgroup of women with chronic pelvic pain. This conclusion is further strengthened by the association between the provoked pain intensity and levels of reported worst pain past week when adjusted for age in a multiple regression analysis. The fact that the highest pain intensities were provoked on the ischial spines and os ischium regions, as compared to the other intra-pelvic structures, indicates that these structures to some extent are an important source of chronic pelvic pain and further investigation is needed to clarify what pathology might explain this.

To the best of our knowledge, no previous study has included the intra-pelvic landmarks used in the present study as a means of evaluating women with chronic pelvic pain. However, in pregnant women, intra-pelvic pain provocation by stimulation of the sacrospinous ligament was one in a combination of tests that best discriminated women with pelvic pain from those without with increased positivity with increased reported pain intensity, in a previous study [27].

Highly prevalent pelvic floor muscle tenderness has been demonstrated in women with chronic pelvic pain, with a lower pain detection threshold as compared with controls [16, 18, 28]. Significant differences of total pelvic floor tenderness were reported between the groups, in those studies, although the sum of scores was generally low [19, 28]. In the present study, the range of sum of scores was wider and from 0 to nearly a maximum score. This supports the use of this method to discriminate those with chronic pelvic pain from those without.

A methodological strength of this study was the blinding procedure in the selection of women without low back or pelvic pain. The examination method used was safe and did not give either of the groups any adverse effects, immediately or delayed. The study has several limitations. One was the non-random selection of study populations which resulted in different mean age between the women of the two groups. However, the association between sum of pain intensity scores and groups remained when adjustment by age was used in the regression analysis. In addition, in a systematic review no association between age and chronic pelvic pain was shown [29]. Lack of information about the presence of co-morbidities that might be involved in the pain mechanism is another limitation. Gynecologic and other visceral causes of pelvic pain should have been excluded although serious illness and visceral pathology were not obvious. A validated pressure algometer device designed for intravaginal examination has been described and successfully used on pelvic floor muscles [18]. By use of such a device the precision in our study could have increased.

In this study, the provoked pain intensity on the different intra-pelvic landmarks was highly correlated with the highest pain intensity reported with pressure on the ischial spine and on the ischium inferior to the ilio-ischial fusion. Despite that provoked pain at the ischial spine was an inclusion criteria, this pattern might be a reflection of the development of the pain, such as the pain might have started in the area of the ischial spines and subsequently has spread out around the pelvic ring. Plausible mechanisms to such a scenario might be biomechanical [30], central sensitization [31], referred pain [32], nerve fiber proliferation or denervation caused by injuries to myofascial pelvic supports succeeded by re-innervation or a combination of these mechanisms [33]. Anecdotally, during the study process, the women frequently reported that the pain provoked by the examination was similar to their chronic pain. This has to be confirmed in future research but could possibly be used in clinical practice to reassure women that this examination indicated a possible source of their enigmatic pain that was not related to visceral pathology.

## Conclusions

Pain provocation by stimulation of anatomical landmarks of the intra-pelvic side-wall could be valuable in a clinical setting to discriminate parous women with pregnancy-related chronic pelvic pain after childbirth from parous women without such pain and to identify a subgroup of parous women with chronic pelvic pain attributable to non-visceral sources. These results need to be confirmed in a larger cohort with different types of chronic pelvic pain and also in subjects with low back pain.

## Abbreviations

CPP: Chronic pelvic pain
SD: Standard deviation

## References

[1] Mathias SD, Kuppermann M, Liberman RF, Lipschutz RC, Steege JF (1996). Chronic pelvic pain: prevalence, health-related quality of life, and economic correlates. Obstet Gynecol.

[2] Grace VM, Zondervan KT (2004). Chronic pelvic pain in New Zealand: prevalence, pain severity, diagnoses and use of the health services. Aust N Z J Public Health.

[3] Berkley KJ, Guilbaud G, Benoist JM, Gautron M (1993). Responses of neurons in and near the thalamic ventrobasal complex of the rat to stimulation of uterus, cervix, vagina, colon, and skin. J Neurophysiol.

[4] Benjamin-Pratt AR, Howard FM (2010). Management of chronic pelvic pain. Minerva Ginecol.

[5] Howard FM (1996). The role of laparoscopy in the evaluation of chronic pelvic pain: pitfalls with a negative laparoscopy. J Am Assoc Gynecol Laparosc.

[6] Warren JW, Morozov V, Howard FM. Could chronic pelvic pain be a functional somatic syndrome? Am J Obstet Gynecol. 2011;205(3):199 e1–5. doi:10.1016/j.ajog.2011.04.003. 10.1016/j.ajog.2011.04.003 21620363

[7] Swanton A, Iyer L, Reginald PW (2006). Diagnosis, treatment and follow up of women undergoing conscious pain mapping for chronic pelvic pain: a prospective cohort study. BJOG.

[8] Laslett M, Aprill CN, McDonald B, Young SB (2005). Diagnosis of sacroiliac joint pain: validity of individual provocation tests and composites of tests. Man Ther.

[9] Hancock MJ, Maher CG, Latimer J, Spindler MF, McAuley JH, Laslett M (2007). Systematic review of tests to identify the disc, SIJ or facet joint as the source of low back pain. Eur Spine J.

[10] Robinson HS, Mengshoel AM, Veierod MB, Vollestad N (2010). Pelvic girdle pain: potential risk factors in pregnancy in relation to disability and pain intensity three months postpartum. Man Ther.

[11] Fall M, Baranowski AP, Elneil S, Engeler D, Hughes J, Messelink EJ (2010). EAU guidelines on chronic pelvic pain. Eur Urol.

[12] Howard FM (2003). Chronic pelvic pain. Obstet Gynecol.

[13] Dalpiaz O, Kerschbaumer A, Mitterberger M, Pinggera G, Bartsch G, Strasser H (2008). Chronic pelvic pain in women: still a challenge. BJU Int.

[14] Apte G, Nelson P, Brismee JM, Dedrick G, Justiz R, Sizer PS (2012). Chronic female pelvic pain--part 1: clinical pathoanatomy and examination of the pelvic region. Pain Pract.

[15] Tu FF, As-Sanie S, Steege JF (2005). Musculoskeletal causes of chronic pelvic pain: a systematic review of diagnosis: part I. Obstet Gynecol Surv.

[16] Fitzgerald CM, Mallinson T (2012). The association between pelvic girdle pain and pelvic floor muscle function in pregnancy. Int Urogynecol J.

[17] Fitzgerald CM, Neville CE, Mallinson T, Badillo SA, Hynes CK, Pelvic TFF (2011). Floor muscle examination in female chronic pelvic pain. J Reprod Med.

[18] Tu FF, Fitzgerald CM, Kuiken T, Farrell T, Harden RN (2007). Comparative measurement of pelvic floor pain sensitivity in chronic pelvic pain. Obstet Gynecol.

[19] Tu FF, Holt J, Gonzales J, Fitzgerald CM. Physical therapy evaluation of patients with chronic pelvic pain: a controlled study. Am J Obstet Gynecol. 2008;198(3):272 e1–7. doi:10.1016/j.ajog.2007.09.002. 10.1016/j.ajog.2007.09.002 18313447

[20] Travell JG, Simons DG. Myofascial Pain and Dysfunction. Lippincott Williams & Wilkins; 1983.

[21] Torstensson T, Lindgren A, Kristiansson P (2009). Corticosteroid injection treatment to the ischiadic spine reduced pain in women with long-lasting sacral low back pain with onset during pregnancy: a randomized, double blind, controlled trial. Spine (Phila Pa 1976).

[22] Carlsson AM (1983). Assessment of chronic pain. I. Aspects of the reliability and validity of the visual analogue scale. Pain.

[23] Salen BA, Spangfort EV, Nygren AL, Nordemar R (1994). The disability rating index: an instrument for the assessment of disability in clinical settings. J Clin Epidemiol.

[24] Vleeming A, Albert HB, Ostgaard HC, Sturesson B, Stuge B (2008). European guidelines for the diagnosis and treatment of pelvic girdle pain. Eur Spine J.

[25] Albert H, Godskesen M, Westergaard J (2000). Evaluation of clinical tests used in classification procedures in pregnancy-related pelvic joint pain. Eur Spine J.

[26] van der Windt DA, Simons E, Riphagen AC II, Verhagen AP, Laslett M, et al. Physical examination for lumbar radiculopathy due to disc herniation in patients with low-back pain Cochrane Database. Syst Rev. 2010;2 CD007431. 10.1002/14651858.CD007431.pub2.10.1002/14651858.CD007431.pub220166095

[27] Kristiansson P, Svardsudd K (1996). Discriminatory power of tests applied in back pain during pregnancy. Spine (Phila Pa 1976).

[28] Montenegro ML, Mateus-Vasconcelos EC, Rosa e Silva JC, Nogueira AA, Dos Reis FJ, Poli Neto OB (2010). Importance of pelvic muscle tenderness evaluation in women with chronic pelvic pain. Pain Med.

[29] Latthe P, Mignini L, Gray R, Hills R, Khan K (2006). Factors predisposing women to chronic pelvic pain: systematic review. BMJ.

[30] Gyang A, Hartman M, Lamvu G (2013). Musculoskeletal causes of chronic pelvic pain: what a gynecologist should know. Obstet Gynecol.

[31] Latremoliere A, Woolf CJ (2009). Central sensitization: a generator of pain hypersensitivity by central neural plasticity. J Pain.

[32] Torstensson T, Butler S, Lindgren A, Peterson M, Eriksson M, Kristiansson P (2015). Referred pain patterns provoked on intra-pelvic structures among women with and without chronic pelvic pain: a descriptive study. PLoS One.

[33] Atwal G, du Plessis D, Armstrong G, Slade R, Quinn M (2005). Uterine innervation after hysterectomy for chronic pelvic pain with, and without, endometriosis. Am J Obstet Gynecol.

